# New Horizons for Phenotyping Behavior in Rodents: The Example of Depressive-Like Behavior

**DOI:** 10.3389/fnbeh.2021.811987

**Published:** 2022-01-05

**Authors:** Hugo Leite-Almeida, Magda J. Castelhano-Carlos, Nuno Sousa

**Affiliations:** ^1^Life and Health Sciences Research Institute (ICVS), School of Medicine, University of Minho, Braga, Portugal; ^2^ICVS/3B’s—PT Government Associate Laboratory, Braga/Guimarães, Portugal; ^3^Clinical Academic Center—Braga, Braga, Portugal

**Keywords:** rodents, welfare, behavior, behavioral paradigms, depression, translational models

## Abstract

The evolution of the field of behavioral neuroscience is significantly dependent on innovative disruption triggered by our ability to model and phenotype animal models of neuropsychiatric disorders. The ability to adequately elicit and measure behavioral parameters are the fundaments on which the behavioral neuroscience community establishes the pathophysiological mechanisms of neuropsychiatric disorders as well as contributes to the development of treatment strategies for those conditions. Herein, we review how mood disorders, in particular depression, are currently modeled in rodents, focusing on the limitations of these models and particularly on the analyses of the data obtained with different behavioral tests. Finally, we propose the use of new paradigms to study behavior using multidimensional strategies that better encompasses the complexity of psychiatric conditions, namely depression; these paradigms provide holistic phenotyping that is applicable to other conditions, thus promoting the emergence of novel findings that will leverage this field.

## Introduction

Mental health and mental health disorders are receiving progressively greater attention from the public. This important recognition is related to the effort of (neuro) scientists and mental health providers to demonstrate its relevance, based on epidemiological data. Amongst psychiatric disorders, depression is a leading cause of disability, affecting about 310 million people worldwide (GHDx, 2021), and is predicted to soon become the second leading cause of the global burden of disease. The underlying causes of depression, and of other complex psychiatric disorders, are not fully understood but the consensual opinion points to an association between genetic/epigenetic and environmental factors (Charney and Manji, 2004; Nemeroff and Vale, 2005; Klengel and Binder, 2013).

Given its multidimensional and heterogeneous characteristics, diagnosing depression poses significant challenges. There is no specific biomarker of depression and diagnosis is based on the guidelines defined in the *Diagnostic and Statistical Manual of Mental Disorders* (DSM-5) or the *International Classification of Diseases and Related Health Problems* (ICD-11); importantly, some of the core symptoms of depression (e.g., anhedonia) are also present in other psychiatric conditions (e.g., Schaub et al., 2021). The list of symptoms also include depressed or irritable mood, cognitive symptoms such as guilt, ruminations and suicidal ideation, emotional symptoms such as anhedonia, neurovegetative symptoms such as abnormalities in sleep, appetite, weight and energy, and psychomotor agitation or retardation (Kennedy, 2008). The simultaneous occurrence of several symptoms over a given period of time increases diagnostic confidence, despite the heterogeneity in clinical presentation, pathophysiology and even treatment response. That reflects a complex clinical pathophysiology that is not mirrored in preclinical models, particularly in the assessment of depressive-like behaviors which is frequently unidimensional. While the importance of these animal models is generally accepted by the research community, their poor predictive power for drug efficacy in humans (among other problems) cannot be ignored (Howe et al., 2018). In this review we discuss the possibilities opened by new caging paradigms allowing 24/7 data acquisition on multiple behavioral dimensions to tackle manifestation of depressive behavior.

## Modeling and Testing Depression in Rodents

Modeling human psychiatric disorders like depression in rodents, in which subjective feelings of worthlessness, excessive or inappropriate guilt, recurrent thoughts of death or suicide, manifest, is challenging if not unrealistic. While such limits translational efforts, relevant information was obtained from preclinical models of depression, especially regarding the molecular and cellular mechanisms underlying the pathology (see for review Krishnan and Nestler, 2008, 2011; Fox and Lobo, 2019; Czeh and Simon, 2021). Indeed, there are many fundamental physiological and behavioral responses that have been evolutionarily conserved between species, and so it is not only legitimate, but also ethically and scientifically responsible, to explore these networks and systems in rodent models of psychiatric disorders (Cryan and Holmes, 2005). This has propelled the development of depression models as well as several behavioral paradigms to screen depressive-like behaviors (see for review Holmes, 2003; Cryan and Holmes, 2005; Sousa et al., 2006; Kalueff et al., 2007; Nestler and Hyman, 2010; Pollak et al., 2010; Wang et al., 2017; Planchez et al., 2019).

There are several strategies to model depression, but so far none is completely satisfactory. When designing a model of a disorder/condition, the most relevant causal events and/or manifestations are normally applied. In depression, stress has long been considered a causal factor, but increasingly, genes that may serve as risk factors are being studied. In fact, and beyond the first attempts to mimic depression with brain lesions (e.g., olfactory bulbectomy; Wang et al., 2007), the most established animal models of depression recapitulate (either in isolation or in a combined mode) such processes through selective breeding (e.g., the Flinder’s sensitive line of rats; Overstreet, 1993) or genetic engineering (see for review Cryan and Mombereau, 2004; Lucki, 2011; Planchez et al., 2019; Scherma et al., 2019), as well as through environmental manipulations—e.g., applying chronic social (Rygula et al., 2005), isolated or combined stressors (Willner, 2005). In the process of internal validation, researchers developing these models have collected data demonstrating that these animals display behavioral endpoints matching the characteristics reported either in the DSM or ICD diagnostic tools as well as neurochemical and molecular features compatible we those observed in clinical settings (Cryan and Holmes, 2005; Kalueff et al., 2007; Markou et al., 2009; Nestler and Hyman, 2010; Pollak et al., 2010). Several studies confirmed the face validity (reproducing symptoms of depression observed in humans), construct validity (the symptoms in the animal should be mediated by equivalent neurobiological mechanisms as in humans) and predictive validity (currently used pharmacological and non-pharmacological treatments for depression should modulate the behavioral changes observed in the animal model) of these models (Willner, 1984; Chadman et al., 2009). In addition to these validation criteria, it is also important to consider reproducibility, reliability and feasibility/usability in across laboratories (Willner, 1997; Pollak et al., 2010); some of these last criteria, have however been quite challenging to be consistently established.

Given the importance of the environment in triggering the most common “exogenous” forms of depression, and in the absence of simple genetic culprits of this disorder, the use of “contextual” models, namely exposure to chronic stress protocols to model depression in rodents, has gained attractiveness in the field. Several experimental exposure protocols have been developed to model depression, including early life stress models (e.g., by maternal separation of young pups for 3 h/day for a period of approximately 2 weeks; Sanchez et al., 2001), social defeat models (Rygula et al., 2005; Krishnan et al., 2007) and chronic mild stress models (Willner et al., 1987, 1992; Willner, 1997, 2005). These models have different, but acceptable, degrees of face, construct, and predictive validity and have been instrumental to improve our mechanistic insights on the neurobiology and treatment of depression (Bessa et al., 2009; Duman, 2010; Nestler and Hyman, 2010; Mateus-Pinheiro et al., 2014).

The description of each of these models has been the subject of several studies, including numerous reviews which will not be repeated here. In contrast, the rationale behind these models will be further analyzed, as it is of interest to examine their ability to measure particular dimensions of depressive-like behavior. The repetition of bouts of social subordination that characterizes *chronic social defeat*, leads to some core symptoms of depression such as anhedonia and social withdrawal, as well as “metabolic syndrome” characterized by weight gain and insulin and leptin resistance (Rygula et al., 2005; Krishnan et al., 2007; Chuang et al., 2010). Alternatively, protocols of daily exposure to unpredictable mild stressors (tilted cages, prolonged light periods, food and water deprivation, wet bedding, cage mate alteration, etc.), randomly presented along a period of at least 6 weeks have also been shown to effective (Willner et al., 1987, 1992; Willner, 1997, 2005). These protocols of unpredictable chronic mild stress (uCMS) combine a less intense social stress component with qualitatively different stimuli that mimic a broader spectrum of environmental challenges to the animal. Obviously, the range of stimuli presented renders the protocol longer, more complex and introduces more confounding factors than those using repeated exposure to a single stressor but importantly, the fact that the exposure to different stressors is unpredictable has a significant benefit to reduce the adaptative response of rodents and, in this way, increase the likelihood of expression of susceptibility to the experimental protocol and the display of depressive-like symptoms. Exposure to uCMS was shown to induce a decrease in responsiveness to rewards in a variety of different behavioral paradigms as well as behavioral despair and learned helplessness, but it also induces several other symptoms including decreased sexual, investigative and self-care behaviors, and decreased locomotor activity during the active period of the light/dark cycle, disturbed sleep patterns—revised in Willner (1997, 2005). Predictive validity of the uCMS depression model was shown by a reversal of depressive-like behaviors by chronic treatment with a wide variety of clinically effective antidepressants (Moreau et al., 1992; Monleon et al., 1995; Forbes et al., 1996; Willner, 1997, 2005; Bessa et al., 2009).

As expected, the effectiveness of the models is assessed by applying testing paradigms to measure outcomes that are of relevance to depression. In fact, similar to the human condition in which questioning the patient about his mood and other symptoms is the keystone of diagnosis, herein the measurements of depressive-like behaviors/phenotypes are critical and should be aligned in the main dimensions reported in the DSM or ICD diagnostic tools (Cryan et al., 2002; Holmes, 2003; Cryan and Holmes, 2005; Matthews et al., 2005; Kalueff et al., 2007; Nestler and Hyman, 2010; Pollak et al., 2010). The most popular behavioral tests of depression in rodents are those measuring unidimensional domains. Those include the *forced swimming test* (FST) (Porsolt et al., 1977) and the *tail suspension test* (TST) (Steru et al., 1985), that capture behavioral despair in rodents through immobility time after exposure to these inescapable stressors. Other *learned helplessness* test, in which animals exposed to unpredictable and uncontrollable stress (e.g., electric foot shock) subsequently develop coping deficits for aversive but escapable situations, is another commonly used test paradigm (Maier and Watkins, 2005). The popularity of these tests relies on the fact that they are inexpensive, easy to use and provide simple and high throughput measures; as an additional virtue, it is possible to establish extensive comparisons with results obtained in other labs and after different manipulations (including therapeutic interventions) (Cryan et al., 2002; Pollak et al., 2010; Castagne et al., 2011). A major drawback, is that these tests provide a reductionist perspective of a complex disorder. Also, there is evidence that floating behavior in the FST could be an adaptive response to a stressor (Mul et al., 2016)—see also for a commentary (Molendijk and De Kloet, 2015)—not necessarily reflecting depression.

As recognized by many clinicians, anhedonia is a core symptom of depression and, therefore, a relevant measure in animal models of depression (Gorwood, 2008). Of course, loss of interest and the inability to feel pleasure and joy cannot be captured in a interrogative way in animals. However, indirect proxies can be obtained in rodents. By far, the most popular is the ***sucrose preference test*** (SPT); as with the behavioral despair and helplessness tests, the SPT is relatively easy to perform, and evaluates the preference for, and consumption of, a sweet/rewarding (sucrose or saccharin) solution in comparison to water/neutral solution (Willner et al., 1987). Several studies reveal that animals submitted to the models described above display a reduction in sucrose preference, that can be reverted by antidepressant drugs, such as imipramine, fluoxetine or citalopram (Papp et al., 1996; Rygula et al., 2006; Bessa et al., 2009). Variations of this test have been developed, such as the Sweet Drive Test (SDT), which integrates food preference measurements with ultrasonic vocalizations (Mateus-Pinheiro et al., 2014). As before, these tests are not immune to criticism, such as the fact that, in most cases, these tests require food-deprivation which might interfere with the physiology and motivation of the animals.

## Limitations in Modeling and Measuring Depressive-Like Behaviors

As discussed above there is a relatively limited set of parameters that can be considered akin to depressive symptoms and that can be objectively measured in rodents, including homeostatic symptoms (e.g., abnormalities in sleep, appetite, weight and energy balance), anhedonia and locomotor behavior (Krishnan and Nestler, 2008; Nestler and Hyman, 2010). Core internalized feelings (e.g., depressed mood, feelings of worthlessness, or excessive guilt) are obviously impossible to infer in rodents. Such misalignment might explain for instance why prototypic antidepressants have been shown to be effective in behavioral despair/learned helplessness tasks upon a single acute challenge—see for a meta-analysis (Kara et al., 2018)—while in clinical settings 2–3 weeks are normally required.

An additional limitation is the periodic, short measurements of specific behavioral dimensions typically used in the field. To circumvent this limitation, several studies make use behavioral tests batteries in sequential days, aiming to target specific behavioral dimensions, as well as their respective neurophysiological biochemical, anatomical or endocrine underpins (e.g., Bessa et al., 2009). However, such strategy carries potential confounders including the sequence of the tests and potential “carry-over” effects. In both cases, the sensitivity of the models to intrinsic and extrinsic factors adds to the normal intra- and inter-laboratory variability. Factors such as sex, age, strain, endocrine, nutritive and social status, and genetics, as well as housing and testing conditions (e.g., time of the day, experimenter, cohort-removal etc.), can impact significantly on depression-related behavioral outcomes (Balcombe et al., 2004; Anisman and Matheson, 2005; Van Driel and Talling, 2005; Castelhano-Carlos and Baumans, 2009; Sorge et al., 2014; Takao et al., 2016) which in turn impact on data reproducibility and on its potential translation. The establishment of consensual standards and the introduction of automated data collection (reducing the human intervention), in adequate, more naturalistic conditions, is essential (see for example Takao et al., 2007). Indeed common housing conditions do not provide satisfactory levels of stimulation diminishing the ethological values of the readout while providing poor animal welfare (Wurbel, 2001; Latham and Mason, 2004; Zhu et al., 2006; Balcombe, 2010); furthermore it can limit the ability to discriminate of depressive-like behaviors (see also below; Castelhano-Carlos et al., 2014). These limitations are of relevance when modeling this condition because the lack of social interactions is not only a trigger of, but also a measure of, depressive behaviors. Moreover, the absence of a complex and “enriched environment also limits the possibility of measuring parameters that may inform on the phenotype of individual animals within a group. Therefore, for both scientific and ethical reasons, animal welfare should be taken into consideration when developing models of psychiatric disorders (Van Der Staay et al., 2009).

To sum up, the testing paradigms referred above have been designed to obtain fast readouts of depressive-like behaviors, normally in contexts of low ethological significance (Cryan and Holmes, 2005; Frazer and Morilak, 2005). These lack relevant temporal information and are prone to technical interferences which can impact on data quality. An urgent reappraisal of the methods and strategies used in the context of preclinical depression research is essential.

## Multimodal Paradigms to Screen Depressive-Like Behaviors

As discussed above, multimodality is a requirement for any new experimental setting destined to address depressive-like behavioral manifestations (possibly extensible to any another modeled neuropsychiatric condition). Such requirement stems not only from the diverse behavioral dimensions that are affect on each model (activity, mood and cognition) but also from the inherent variability observed even in inbred colonies—see example below (Torquet et al., 2018). Such variability can simply be the result of differences in the phenotypical manifestation of depression—i.e., subjects are equally affected by the depression-inducing manipulation (e.g., stress) but manifest it in various manners—but can also result from different susceptibilities to the modeling condition between subjects—e.g., stress; see for instance (Magalhaes et al., 2017, 2018, 2019). A second aspect that needs to be considered is the setting itself is how it can impact in the quality of the obtained data. For instance, in an unenriched vs. enriched housing comparison it was found that the former presented enhanced sensitivity to reward loss in the absence of any additional manipulation (Burman et al., 2008; see also for review Rogers et al., 2019; Smail et al., 2020). Also, evidence gathered in studies using enriched environments where rodents’ face more challenges than standard rodent housing, in a naturalistic context has the potential to produce more consistent and valid results. Our series of studies on the PhenoWorld (PhW) provide some evidence in this direction (Castelhano-Carlos et al., 2014, 2017). In a comparison between a PhW colony of rats and rats housed in standard cages (6 rats in both cases) a number of aspects emerged: PhW animals had (i) better circadian sleep/wake rhythms in comparison to standard housed animals; (ii) reduced/increased levels of corticosterone at light/dark phases; and (iii) better performance in helplessness, anhedonia and anxiety paradigms. Importantly, the PhW was able to detect behavioral alterations (reduction in food consumption and running wheels) induced by chronic mild stress as well as recovery upon antidepressant treatment. As potential factors contributing to these results are the large areas [animals can perform more species-specific natural behaviors) and the availability of enrichment elements (running wheels), a certain controllability over the housing environment (access to the different areas is RFDI (radio frequency identification) controlled] and reduced contact with the experimenter—see for an example of experimenter influence (Sorge et al., 2014). Thus, the PhW naturalist environment aligns the concept that “better animal models ensure more generalizable results” (Poole, 1997). A third aspect pretrains with the ability to collect longitudinal strings of data and to maintain a continuous control over the setup. This is of particular interest in the context of depression, as the dysregulation of activity cycles can provide a good readout of maladaptive behavior (e.g., Shimizu and Hara, 2020; Yuan et al., 2020; Li et al., 2021; see also for review Mendoza and Vanotti, 2019). Moreover, it has been demonstrated that the temporization of specific behaviors to specific time periods (e.g., food availability/activity period) can be beneficial regarding the manifestation of depressive- and anxiety-like behaviors (Guerrero-Vargas et al., 2021); such can be easily achieved in the PhW which again can contribute to unmask depression triggering factors by lowering baseline depressive-like behaviors.

Setups like the PhW also permit use of standard behavioral tests in an automated way, by monitoring access to the test arenas (spontaneous exploration). Thus, impressive data sets can be collected for individual animals and opens the way to predict individual trajectories that are of relevance to better phenotyping. Besides facilitating correlations/associations, including with genotyping, these combinatorial measures are likely to lead to the discovery of novel mechanisms that underly depressive-like behaviors and, likewise, of better interventions that may eventually be tailored to individual profiles of the disorder. Although costs involved are relevant, the possibility of gaining a holistic view of behavioral changes, represents a unique opportunity to leverage the quality of the behavioral data produced; ultimately, one might envisage that a single longitudinal observation of a group of animals might produce behavioral data relevant from multiple behavioral dimensions which is of relevance for complex neuropsychiatric disorders.

Moreover, and given that several other layers of complexity in the assessment of different behavioral domains can be added in the PhW and that the analysis of longitudinal variations of these measurements is yet to be performed, it is reasonable to assume that these paradigms have the potential to add valuable insights to phenotypic characterization of mood disorders in animal models. For instance, the PhW paradigm permits the simultaneous characterization of the cognitive domain of the animals which is another behavioral domain affected in depression and shown to present with mood and anxiety behavioral alterations in a comorbid manner (Bessa et al., 2009). Or, by analysis of operant behavior available in the PhW, assessment of value reward and decision-making may be incorporated to phenotyping analyses of depressive-like behavior (Der-Avakian et al., 2013; Morgado et al., 2015). The observation of increased social interaction of rats living in the PhW in comparison to standard housed animals allied to the performance of natural species-specific behaviors such as gnawing, climbing, running and hopping, grooming, constitute evidence that, even though in the laboratory context, the PhW creates a more naturalistic environment for the animals. Combining this fact with the automated testing of animals avoids important interferences from external factors that imply intra- and inter-laboratory variability and promote a more realistic analysis of trait factors (Balcombe et al., 2004; Anisman and Matheson, 2005; Van Driel and Talling, 2005; Willner, 2005; Castelhano-Carlos and Baumans, 2009; Sorge et al., 2014). Variability of experimental results performed in such conditions will reflect individualities of each animal, which are valuable for the translation of results obtained with animal models into the human situation, contributing to progress in the knowledge of the complex brain-behavior processes in health and in disease. In this way, the PhW constitutes a good setting to analyze inter-individual relations in a group.

Other commercially available experimental settings like the LABORAS, PhenoCube or the IntelliCage also offer interesting solutions. The latter is probably one of the most used alternative in mice—see for a recent review (Kiryk et al., 2020)—including for the assessment of depressive-like behaviors (e.g., Branchi et al., 2010; Cathomas et al., 2015; Milior et al., 2016; Alboni et al., 2017; Oizumi et al., 2020; Sun et al., 2021)—particularly anhedonia. These solutions are also highly versatile, i.e., they accommodate many experimental designs, and contrary to the PhW are relatively small. However, a great advantage of settings like the PhW is its modular nature as different paradigms can be easily incorporated in the central structure. For instance, Torquet et al. (2018) redesigned it and associated a T-maze to test for decision-making—“Souris City”—to demonstrate in a relatively homogenous inbred mice population individual social and cognitive differences as well as the underlying neurobiological substrates. Finally, another front advancing fast in behavioral analysis comes from the application of machine learning approaches to the analysis of 2D videos but also 3D tacking advantages of multiple sources and triangulation techniques—see a recent comment here (Vogt, 2021) and references within. These offer excellent perspectives for the analysis of social behaviors and other behavioral dimensions also affected in mood disorders.

## Conclusion

Herein we have reviewed the most relevant aspects of modeling and phenotyping depressive-like behavior in rodents. A brief overview of the available, and more popular models and tests was given highlighting their strengths and limitations. Using the PhW as an example, we reviewed the benefits of using novel paradigms that leverage the possibilities of phenotyping across multiple dimensional domains either at individual or group levels with unprecedent precision and quality that may carry this area of research (and certainly others) to higher standards and novel horizons.

## Author Contributions

NS conceived the idea. MC-C and HL-A prepared a draft. All authors contributed to the final version.

## Conflict of Interest

The authors declare that the research was conducted in the absence of any commercial or financial relationships that could be construed as a potential conflict of interest. The handling editor is currently organizing a Research Topic with one of the authors NS.

## Publisher’s Note

All claims expressed in this article are solely those of the authors and do not necessarily represent those of their affiliated organizations, or those of the publisher, the editors and the reviewers. Any product that may be evaluated in this article, or claim that may be made by its manufacturer, is not guaranteed or endorsed by the publisher.
